# Home Language Experience Shapes Which Skills Are Used during Unfamiliar Speech Processing

**DOI:** 10.3390/languages9050159

**Published:** 2024-04-26

**Authors:** Susannah V. Levi

**Affiliations:** Department of Communicative Sciences and Disorders, New York University, New York, NY 10012, USA

**Keywords:** spoken word recognition, working memory, linguistic experience, ease of language understanding model

## Abstract

Speech mixed with noise and speech that is of an unfamiliar variety both make the task of understanding more difficult. Children are often more negatively affected by these situations than adults. Numerous studies have examined the cognitive and linguistic skills that support spoken language processing. In the current study, we examine the contribution of linguistic exposure and various cognitive and linguistic skills for spoken word recognition of an unfamiliar variety of speech (German-accented English). The Ease of Language Understanding model predicts that working memory skills are needed in the most difficult listening situations. Two groups of school-age children were drawn from a larger sample: those with exposure to multiple languages in the home and those exposed to only English in the home. As predicted, working memory skills predicted performance for children with less varied linguistic experience (those only exposed to English in the home), but not for children with varied linguistic exposure. In contrast, linguistic skills predicted performance for children with more varied linguistic experience, even though the two groups did not differ overall in any of the assessed skills. These findings support the Ease of Language Understanding model of language processing.

## 1. Introduction

When processing spoken language, humans often find themselves listening in nonoptimal, noisy environments. Listening to speech in these settings is difficult, as acoustic properties of the signal can be masked, making the speech less intelligible to listeners. Listening in noise is especially difficult for children (Erickson and Newman 2017; Leibold and Buss 2019). More recent research on spoken language processing has examined not only bottom-up (signal-level) aspects, but also various top-down aspects (e.g., linguistic context) and listener-level characteristics (e.g., vocabulary size, working memory) that affect accuracy of perceiving speech. The Ease of Language Understanding model (Rönnberg et al. 2013, 2019; Stenfelt and Rönnberg 2009; Rönnberg 2003) incorporates all of these levels of information to predict for which listeners and under what conditions spoken language processing is more or less successful. The model further predicts which skills are required during different listening conditions. In particular, the model predicts that working memory is relied on when the listening task is more difficult. In the current study, we examine the extent to which various cognitive resources are recruited during spoken word recognition and how linguistic exposure during childhood (children exposed to another language in the home versus those who are exposed to only the single, local variety of the language) affects which cognitive resources are utilized when processing an unfamiliar variety of the language, in this case, German-accented English.

### 1.1. Variation in Spoken Word Recognition Accuracy among Children

Children have more difficulty processing speech in the presence of noise than adults (Erickson and Newman 2017; Leibold and Buss 2019), though this ability improves across childhood (Munson 2001; Eisenberg et al. 2000; Elliott 1979; McCreery et al. 2020; Bent 2014). One explanation for why children perform worse than adults and why they improve with age is that younger children have less linguistic knowledge, often measured through vocabulary size or language ability (typically measured through standardized assessments that examine morphosyntactic and syntactic processing). Vocabulary scores have been found to predict spoken word recognition among children with typical development (Munson 2001; Klein et al. 2017; Bent 2014) and those with hearing aids (Klein et al. 2017), though the impact of vocabulary may only emerge with less familiar words (Klein et al. 2017). Among autistic children, vocabulary size has been found to predict spoken word recognition but does not account for unique variance once age is accounted for (Venker 2016). For sentence recognition tasks, studies have found effects of vocabulary to be stronger among younger children (5–6 year olds) than for older children (9–10 year olds) (McCreery et al. 2020; Miller et al. 2019). Together, these studies indicate that larger vocabularies may help children process spoken language, though this may only happen below a certain age or in more difficult tasks (e.g., with less familiar words).

Other studies have examined how differences in language ability affect spoken language processing tasks. In several studies, language ability has been treated as a binary variable, by dividing children into two groups: those with typical language development (TLD) and those with developmental language disorder (DLD). Language ability is typically determined through various standardized assessments and can include sentence recall and other tests of morphosyntax. For example, Mainela-Arnold et al. (2008) used a gating task in which children heard successively longer portions of words and guessed the word. Although the DLD and TLD groups did not differ in the amount of acoustic information needed to initially select the correct word, the children with DLD continued to respond with incorrect words at successively longer gates, whereas children with TLD settled on the correct target word with less acoustic information. In another study, Dollaghan (1998) did not find differences between children with and without DLD in spoken word recognition using a gating task for words that were familiar, but did find that children with DLD performed worse than those with TLD for unfamiliar words. Using a visual world paradigm with adolescents, McMurray et al. (2010) found that language ability predicted performance, especially at later stages of processing. Similar to the studies examining vocabulary on spoken word recognition, there is some evidence that language ability does not influence performance for easier words (e.g., highly familiar items) (Montgomery 1999; Dollaghan 1998).

Not all studies that have examined linguistic skills have found that they predict spoken word recognition. For example, Evans et al. (2018) did not find overall differences between children with and without DLD on a spoken word recognition task using a gating paradigm but did find that different cognitive measures predicted performance in the two groups. In particular, inhibition and shifting significantly predicted spoken word recognition in children with DLD, whereas receptive vocabulary size and updating predicted performance in the children with TLD. The study suggests that even in cases where the overall performance appears the same (in this case, similar scores on the spoken word recognition task), different groups of children tap into different skills to perform the task. Thus, it is important to explore the impact of cognitive and linguistic skills among groups of children and how they variably predict performance on spoken language tasks.

In addition to examining linguistic abilities and their impact on spoken word recognition, numerous recent studies have examined the contribution of other cognitive skills on spoken word recognition. As mentioned above, Evans et al. (2018) examined a variety of cognitive skills related to spoken word recognition. One skill that has been examined is working memory. The specific model of working memory varies across studies, in particular, in how different components of working memory are divided and defined. Broadly speaking, working memory involves temporary storage of information (verbal or nonverbal), manipulating or analyzing that information, and interfacing with long-term memory representations (Baddeley and Logie 1999; Baddeley 2000, 2003; Cowan 2017, 1988). McCreery et al. (2017) examined spoken language processing in a variety of contexts including spoken word recognition. For this task, they did not find that higher vocabulary scores were associated with better spoken word recognition once age was accounted for; however, higher working memory was associated with better performance. Using a sentence recognition task, McCreery et al. (2020) found that working memory predicted performance among both older and younger children, but only in the most difficult masking condition.

Taken together, the studies reviewed in this section show that both linguistic skills and other cognitive skills (e.g., working memory, executive function) often contribute positively to spoken language processing. Importantly, these studies also show that the contribution of these individual skills may depend on the task difficulty (e.g., high- versus low-familiarity words) and that groups of children may vary in which skills are tapped into.

### 1.2. Differences in Spoken Language Recognition Based on Linguistic Experience

Linguistic experience, especially exposure to linguistic variability, has often been found to affect spoken word recognition or spoken sentence recognition. Using a lab-based exposure paradigm and a sentence recognition task, Baese-Berk et al. (2013) found that adults exposed to multiple varieties of accented speech performed better with a novel, unfamiliar accent than those who were not exposed to varied accents during training. Potter and Saffran (2017) tested US infants (18 months) listening to an unfamiliar variety of English (British English). One group of infants was given brief exposure to several varieties of English, and a second group was given exposure to a single variety (British English) from multiple speakers. Only the infants who were exposed to multiple varieties of English were able to recognize English words produced by a British speaker, suggesting that exposure to varied input can be an advantage for young children. In a word learning task with toddlers, Kartushina et al. (2022) found that toddlers raised in bi-dialectal homes were better than those in mono-dialectal homes at learning words presented in multiple dialects, again suggesting that exposure to multiple varieties of a language in the home facilitates processing.

Despite some evidence for the benefits of varied linguistic exposure, not all studies have demonstrated these benefits. Using meaningless sentences, Levy et al. (2019) examined accuracy for German-speaking children with different types of linguistic exposure at home. Sentences were produced by a native speaker of the local variety of German, a speaker of an unfamiliar dialect of German, and by a speaker whose first language was Korean. Statistical analyses included experience with other languages, experience with speech produced by second language users of German, and experience with other dialects of German as continuous predictors. Neither experience with other languages nor experience with speech produced by second language users of German predicted accuracy in any conditions. Interestingly, experience with other dialects of German did result in better sentence recognition accuracy. Taft and Tao (2017) examined spoken sentence recognition of speech produced by a speaker of an unfamiliar accent by three groups of adult listeners: those exposed to accented speech at home, those exposed mostly to a different language in the home, and those exposed only to the local variety of English in the home. The adults exposed to only the local variety in the home performed better than the two other groups when transcribing sentences from a speaker with an unfamiliar accent. In other words, the adults who had been exposed to more varied speech at home during childhood actually performed worse. It is worth noting that the groups differed on a variety of other measures such as vocabulary, parental education, and nonverbal intelligence, with the speakers exposed to a different language at home having lower scores. In another study, Miller et al. (2019) examined sentence recognition in younger (5–6 years) and older (9–10 years) children who were either monolingual English listeners or bilingual Spanish-English listeners. They found that the bilingual children performed worse on the sentence recognition task, but these children also had lower vocabulary scores. When vocabulary was entered into the model, linguistic exposure was no longer a significant predictor.

Taken together, these studies provide some evidence for the benefits of varied linguistic exposure for spoken language processing tasks, though the findings are mixed. In some instances (e.g., Miller et al. 2019), negatives seem to exist, though these can be attributed to other group differences (e.g., vocabulary). Thus, these studies demonstrate the importance of ensuring other cognitive, linguistic, and social factors are minimized across groups.

### 1.3. Ease of Language Understanding Model

The studies reviewed in the previous sections demonstrate that various cognitive and linguistic skills, as well as linguistic experience, affect spoken language processing. The Ease of Language Understanding model (Rönnberg et al. 2013, 2019; Stenfelt and Rönnberg 2009; Rönnberg 2003) is a model of language processing that incorporates many of these factors. In particular, the Ease of Language Understanding model outlines how and when factors related to the stimuli (bottom-up factors) and those that relate to individual differences in experience or cognitive and linguistic skills (top-down factors) affect performance on spoken language tasks. The model incorporates aspects like working memory and executive function and makes explicit predictions about the circumstances under which various skills are more likely to be utilized during language processing. In the model, bottom-up, signal-related processing is considered to happen automatically and tap into a listener’s ability to map incoming acoustic information to stored representations. When the signal is degraded (e.g., from the addition of noise) or differs from the ambient speech or the speech experiences of the listener, then top-down processes must be recruited to accurately understand the incoming speech. Thus, the model predicts that under more difficult listening situations or in the presence of mismatches between incoming speech and long-term memory representations, the effect of individual differences for various top-down skills (in particular, working memory capacity and executive function) will be revealed. In addition, the model predicts that as listeners become more familiar with these different types of speech, listeners will rely less on these cognitive skills (Rönnberg et al. 2019). Similarly, the more predictive utterances are, the less reliance there is on cognitive skills (e.g., high predictability sentences) (see Akeroyd 2008 for a review). Under this framework, understanding individual words provides the least amount of semantic support and, thus, should more likely require reliance on working memory skills.

Several studies with children provide support for this model. McCreery et al. (2017) found that higher working memory skills predicted speech recognition in noise across different types of stimuli including words, semantically anomalous sentences, and typical sentences. Blomberg et al. (2019) examined spoken sentence processing in children with and without attention deficit hyperactivity disorder, based on previous work indicating that these children have poorer working memory and selective attention skills. Using a composite score of working memory and selective attention, they found that children with better cognitive skills had higher accuracy when processing difficult (noisy or distorted) speech.

### 1.4. The Current Study

The current study examines spoken word recognition by school-age children. In particular, we examine the effects of linguistic experience, lexical familiarity, and various individual differences measures of linguistic and other cognitive skills. In terms of linguistic experience, children in the current study are grouped based on whether they have varied linguistic exposure (see Section 2.1 for more information). Words are split into two groups, high familiarity and low familiarity. Because the items are individual words, which lack contextual semantic support, children are more likely to tap into different cognitive skills to complete the spoken word recognition task. Furthermore, the items are produced by German L1–English L2 speakers who use a variety of English that is unfamiliar to all of the children, making it more likely that children will need to tap into cognitive skills to optimally complete the task. of English. Based on the Ease of Language Understanding model, we can make several predictions about when children will tap into various cognitive skills.

1. Children with exposure to only one variety of English (single-variety exposure group) are expected to rely more on cognitive skills (especially the short-term memory component of working memory) than children who are exposed to multiple languages or varieties of English in the home (multiple-variety exposure group).
2. Working memory skills are expected to predict performance more strongly for low-familiarity words compared to high-familiarity words.
3. Older children are expected to perform better than younger children.

## 2. Methods

### 2.1. Participants

Fifty-eight children, ages 6;8–11;9, were selected from a larger study conducted in the lab. All children were English-dominant, living in New York City at the time of testing, and attended schools with instruction only in English. To be selected for inclusion in the current study, children had to have completed the baseline spoken word recognition task, as well as all of the other individual differences measures described below.

Group membership was determined by the parent report. Parents were asked to indicate which language(s) their child (i) spoke, (ii) used most, (iii) used best, (iv) preferred, and (v) understood best. In addition, the parent report asked for information about all the people living in the home and what languages they spoke, and also if there was any other exposure to another language through childcare (e.g., through a babysitter). Children were included in the single-variety exposure group if English was the only language reported on all of these questions. Children for whom the parent report indicated an additional language on the *what languages does your child use* questions or indicated that there was exposure to another language in the home or childcare were included in the multiple-variety exposure group. The additional languages from the children were Arabic, Catalan, Chinese, French, Fulani, Greek, Hindi, Japanese, Mandingo, Russian, Spanish, Tagalog, Ukrainian, and Wolof (note, these are the language terms used on the parent report). The multiple-variety exposure group had 25 children (10 female, 15 male), and the single-variety exposure group had 28 children (13 female, 15 male). At the time these data were collected (2008–2014), only two options were given on the parent report: male, female. None of the included children had any exposure to German or German-accented English based on the parent report. Given that some previous studies did not control for group differences in measures of language and cognition, we conducted t-tests to confirm that the groups of participants did not differ. Descriptive statistics and group comparisons are presented in Table 1. All children also passed a hearing screening. This study was approved by the Institutional Review Board at New York University. Parents gave consent, and children gave assent to participate.

### 2.2. Materials for the Spoken Word Recognition Task

Three hundred sixty monosyllabic consonant–vowel–consonant (CVC) English words were produced by six female German-English bilingual speakers from Germany (excluding southern Germany). Additional information about the recordings and speakers can be found in Levi et al. (2007). In brief, all speakers were adults below the age of 30 and were living in the US for 1–4 years at the time of the recording. Age of acquisition ranged from 9 to 13 years. Words were split into those that were likely to be highly familiar to children in this age range and those that were likely to be unfamiliar to children in this age range. The familiarity rating was determined by two speech-language pathologists and the author. Only items that were agreed upon by all three raters were included as low (109 words) or high (125 words) familiarity. See Levi (2015) for additional details about how the items were classified into the two familiarity bins. From these words, different subsets of 48 items (24 high familiarity and 24 low familiarity) were selected for the spoken word recognition task.

### 2.3. Procedure

Children who participated in the larger study completed a spoken word recognition task on the second day of the study and completed the other standardized assessments and experimental tasks across other days of the study, which spanned 7–12 sessions. The order and day of the other assessments and tasks varied depending on how much time children were available for the testing. For more information about the full protocol, see Levi (2015).

#### 2.3.1. Spoken Word Recognition

The spoken word recognition task consisted of 48 CVC words evenly distributed across the six speakers and the two familiarity bins. Children were given a practice block with eight additional words produced by a seventh German-English bilingual speaker to familiarize them with the task and noise level. All items were mixed with signal-dependent noise (Schroeder 1968; Benkí 2003; Felty 2007) and presented at 5 dB signal-to-noise level. Items were presented at a comfortable level and presented over circumaural headphones. Children said the words they heard aloud and were asked to make their best guess if they were unsure. A trained research assistant transcribed the items live, and a recording was also made for transcription by a second trained research assistant. Any discrepancies in transcription were resolved by a third transcriber. For the current study, items were scored as whole word correct.

#### 2.3.2. Assessments of Linguistic Skills

The Recalling Sentences subtest of the Clinical Evaluation of Language Fundamentals 4th edition (CELF-4: Semel et al. 2003) was used as a proxy for children’s language ability, similar to other sentence repetition tasks that have been used for differentiating children with and without a language disorder (Redmond 2005; Redmond et al. 2011). In the Recalling Sentences task, children hear sentences that increase in length and complexity and are asked to repeat them back. Raw scores were converted to standard scores by age, which have a mean of 10 and a standard deviation of 3. As a measure of receptive vocabulary, children were administered Form B of the Peabody Picture Vocabulary Test (PPVT: Dunn and Dunn 2007). In this task, children hear a word, see four pictures, and are asked to indicate which picture matches the word. Age-normed scores on the PPVT have a mean of 100 and a standard deviation of 15. Children also completed the Blending and Elision subtests of the Comprehensive Test of Phonological Processing (CTOPP: Wagner et al. 1999), which combine to form the Phonological Awareness composite score. The Blending task asks children to take sounds or syllables and combine them into a word (e.g., *What word do these sounds make? can-dy*). For the Elision task, children are asked to take a sound out of a word and say what word remains (e.g., *Say time without saying [t]*). This composite has a mean of 100 and a standard deviation of 15. For children who completed all subtests of the CELF-4 that create the Core Language score, these were used to examine group differences but not for the statistical analyses related to the spoken word recognition task, because one child did not complete all of the necessary subtests. The Core Language scores have a mean of 100 and a standard deviation of 15.

#### 2.3.3. Assessments of Cognitive Skills

The ELU model refers to working memory capacity and executive functioning skills as important cognitive skills that influence spoken language processing in conditions that are more difficult (e.g., with noise). Within the model, working memory capacity, referred to as RAMBPHO (Rapid, Automatic, Multimodal Binding of PHOnology), is often tested with a complex reading span task in which children read sets of sentences rapidly, complete a task that ensures their comprehension of each sentence, and then recall the first or last word of each sentence in the set. In the current study, Baddely’s model of working memory (Alloway et al. 2004; Pickering and Gathercole 2001), which parses out various components of storage and manipulation of information, is used exactly because it separates out various skills that make up the working memory system. In addition, Baddeley’s model, along with the tasks thought to tap into each component, is used because previous research has found that these components differentially affect other types of auditory processing (Levi 2014).

Children completed a forward digit span task, which is thought to tap into the phonological loop component of Baddely’s model and represents short-term memory storage and taps into verbal rehearsal (Alloway et al. 2004; Pickering and Gathercole 2001). In the task, children hear a set of numbers and must repeat them back in the same order. Two lists at each length are presented starting with length two. The backward digit span task, which taps into the central executive component of Baddely’s model (Alloway et al. 2004; Pickering and Gathercole 2001), was the same as the forward task, except that children were asked to repeat the numbers back in the reverse order. The backwards digit span task requires not only short-term memory storage, but also manipulation of items. Both of the digit span tasks are age-normed as part of the CELF-4. These subtests have a mean of 10 and a standard deviation of 3. The block span from the Children’s Working Memory Test Battery (Pickering and Gathercole 2001) is a nonverbal analog to the forward digit span and taps the visuospatial sketchpad, which is the short-term memory store for nonverbal information. The experimenter points to grey blocks randomly arranged on a board, and the child is instructed to point to the blocks in the same order. The scaled scores from the block span have a mean of 100 and a standard deviation of 15.

### 2.4. Statistical Analysis and Coding

For the spoken word recognition task, words were scored as whole word correct (1) or incorrect (0). First, an omnibus logistic mixed-effects model was created in R using *lme4* (Bates et al. 2010) that included fixed effects for group (multiple-variety, single-variety exposure), lexical familiarity (high, low), block (first, second), age, Recalling Sentences, PPVT, Phonological Awareness, Forward Digit Span, Backward Digit Span, Block Span, and an interaction between group and lexical familiarity. The model also included random intercepts for participant, item, and talker. All categorical predictors were sum-coded, and all continuous predictors were scaled and centered. This model was created to examine group differences and whether the groups differed for the high- versus low-familiarity items. Scaled scores were used for all of the individual differences measures.

Given previous studies demonstrating that individual differences in linguistic and cognitive measures may be tapped into for different groups, even in the absence of overall group differences (Evans et al. 2018), and given the predictions of the Ease of Language Understanding model that the more difficult items may be more likely to require tapping into working memory, four additional models were created with the same fixed and random effects as the omnibus model on the following four subsets of data: high-familiarity items for the multiple-variety exposure group, low-familiarity items for the multiple-variety exposure group, high-familiarity items for the single-variety exposure group, and low-familiarity items for the single-variety exposure group. In addition, Pearson correlations were conducted among the individual differences measures to ensure that no variables were highly correlated.

## 3. Results

Pearson correlations between age and all working memory and linguistic measures were conducted on the scaled scores in R using the *Hmisc* package version 5.1–2 (Harrell 2024). These are presented in Table 2. The majority of correlations were weak to moderate, though it must be noted that cut-offs vary for what constitutes moderate (Cohen 1988; Akoglu 2018; Schober et al. 2018). The three linguistic measures (PPVT, Recalling Sentences, Phonological Awareness) were all moderately correlated. In addition, Recalling Sentences was moderately correlated with the Forward Digit Span, which is not surprising, given that the Recalling Sentences task taps into verbal rehearsal skills, as does the Forward Digit Span task.

**Figure 1.**
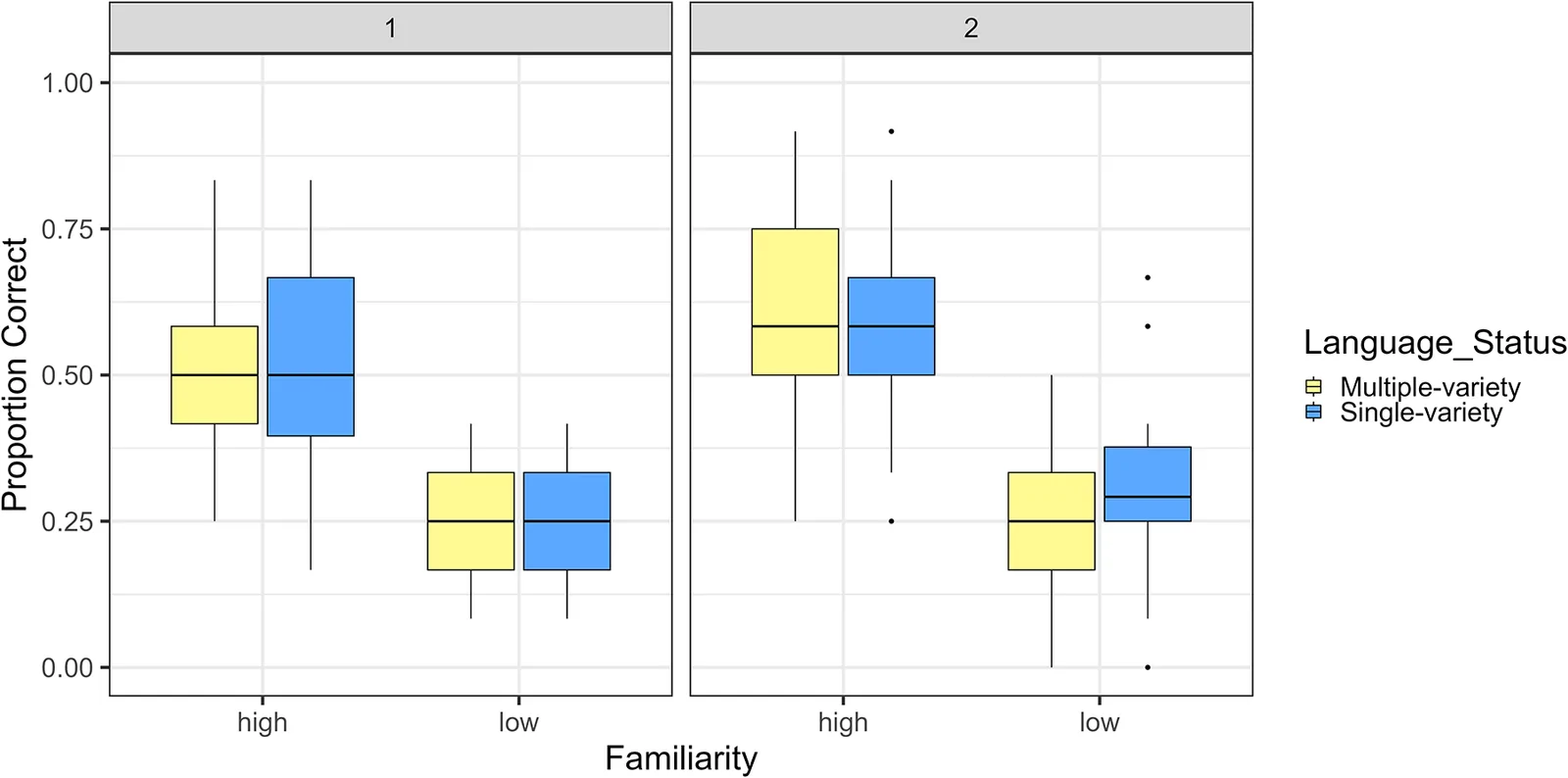
Boxplot proportion correct of spoken word recognition for group by lexical familiarity, faceted by block.

The omnibus model only revealed significant effects of familiarity (*p* < 0.001), block (*p* = 0.001), and age (*p* < 0.001) but not of group. As expected, children were better on high-familiarity items than low-familiarity items and better on the second block than the first block (see Figure 1). Also as expected, older children performed better than younger children. In addition, the model revealed a significant effect for the two verbal working memory measures, forward digit span (*p* = 0.034), and backward digit span (*p* = 0.021). Children with higher scaled forward digit spans had higher word recognition scores. Surprisingly, the reverse was true for backward digit span, where children with higher scaled backward digit spans had lower word recognition scores. No other predictors reached significance. The full model output is provided in Appendix A.

Given the moderate correlations between some of the predictor variables, both the variance inflation factor (vif) and the condition number (kappa) were calculated to check for collinearity (Cohen et al. 2003). The conventional cut-offs are 10 for vif and 30 for kappa. All vif values were less than or equal to 2.65, and kappa was 3.51 for the omnibus model.

The four additional models differed in which measures predicted performance. The full model outputs are provided in Appendix A. For the multiple-variety exposure group and high-familiarity items, the model revealed significant effects for block (*p* = 0.049) and recalling sentences (*p* = 0.029). As expected performance was better on the second block compared to the first, and children with higher scaled scores for recalling sentences performed better on the word recognition task. No other predictors reached significance. All vif values were less than or equal to 4.22, and kappa was 6.08. For the multiple-variety exposure group and low-familiarity items, the model only revealed a significant effect of recalling sentences (*p* = 0.043) in the expected direction. No other predictors reached significance. All vif values were less than or equal to 4.43, and kappa was 6.07.

For the single-variety exposure group and high-familiarity items, the model revealed effects for age (*p* < 0.001), forward digit span (*p* = 0.001), and backward digit span (*p* = 0.006). Both the age and forward digit span effects went in the expected direction: older children performed better than younger children, and children with higher scaled forward digit span scores performed better than those with lower forward digit span scores. Like the omnibus model, the backward digit span had a negative impact on performance with higher scores associated with lower performance on the word recognition task. No other predictors reached significance. All vif values were less than or equal to 2.80, and kappa was 4.43. Similarly, for the single-variety exposure group and low-familiarity items, the model revealed effects for block (*p* = 0.022), forward digit span (*p* = 0.010), and backward digit span (*p* = 0.019). Both block and forward digit span scores had positive effects on spoken word recognition, and the backward digit span had a negative effect. No other predictors reached significance. All vif values were less than or equal to 2.67, and kappa was 4.44.

## 4. Discussion and Conclusions

The current study examined spoken word recognition of German-accented (unfamiliar) speech by children. Of particular interest was whether different linguistic experiences would result in different performance on the task. No group-level differences were found between the multiple-variety exposure children—those who were exposed in the home to additional languages—and the single-variety exposure children—those who were only exposed to the ambient local variety of English. Despite a lack of overall group differences in accuracy, additional analyses revealed that the two groups of children tapped into different skills to perform the task. These results are largely consistent with the predictions made from the Ease of Language Understanding model.

Previous studies of spoken word recognition in children have often found that measures of linguistic skills (vocabulary scores or language ability) predict performance on spoken word recognition tasks, though this relationship may be limited to less familiar words (Klein et al. 2017; Dollaghan 1998). In the current study, language ability (as measured by the recalling sentences task) was only predictive of performance for the children from the multiple-variety exposure group, and interestingly, age was not predictive. Recall that the scores entered in the models were normed/scaled scores. Thus, we can interpret these findings as indicating that regardless of age, children with better language ability (higher scaled scores on the recalling sentences task) performed better on the spoken word recognition task. The significant effect of recalling sentences was found for both high- and low-familiarity items and thus was not limited to only those items that may have been more difficult to process. A similar pattern of results was not found for the children in the single-variety exposure group. In fact, for that group of children, no linguistic skills predicted performance. Some previous studies also showed no significant effect of linguistic skills on spoken language processing (Evans et al. 2018). It is worth noting that vocabulary size, one of the most common measures used in previous studies, did not predict performance in any of our models. This type of finding is also not without precedent; McCreery et al. (2017) also did not find an effect of vocabulary once age was accounted for (though it must be noted that they used raw scores not scaled scores). Instead, McCreery et al. (2017) did find that working memory predicted performance (see below for more discussion).

A primary goal of the current study was to examine how linguistic experience impacts spoken word recognition. The omnibus model did not reveal any group differences; in other words, children performed similarly regardless of the linguistic environment in the home. It is important to note that the current study included two groups of children who did not differ in the other linguistic and cognitive measures, a problem that affected previous studies of group differences. Despite the lack of group differences, the follow-up analyses did reveal that the groups differed in which skills predict performance. The Ease of Language Understanding model predicts that working memory skills will be used more when the task is more difficult. This is the case for items mixed with noise versus in quiet (not tested in the current study), for listeners with less experience with the type of speech, and when the items have less predictability or are less expected (low-familiarity items). Evidence in support of the Ease of Language Understanding model comes from the difference outcomes for the models for the two groups of children. For the children with less experience with accented speech (those in the single-variety exposure group), forward digit span predicted performance for both high- and low-familiarity items. For children with more experience with linguistic variability (those in the multiple-variety exposure group), the expectation was that listening to an unfamiliar variety of speech would be less different from stored representations, making the process of understanding spoken language easier, without the need to tap into working memory. Indeed, the mixed-effects models for these children did not reveal an effect of working memory. Contrary to our expectations, we did not find that low-familiarity items were more likely to tap into working memory skills. It is important to point out that the differences in which cognitive and linguistic skills predict performance cannot be attributable to overall group differences in these measures. Importantly, the two groups of children were matched on all of the relevant predictors.

The final prediction of the current study was that older children would perform better on the task than younger children. Overall, this was found to be the case when all children were pooled in a single model. However, this pattern was only found for the single-variety exposure children once the groups were separated. In contrast, for those exposed to variable linguistic input, overall language ability (as measured by recalling sentences) was the sole individual difference measure to be significant.

One unexpected finding emerged from the study: for children in the single-variety exposure group, better backward digit span scores were associated with *poorer* spoken word recognition. This task is used to tap into the central executive component of Baddeley’s model of working memory (Alloway et al. 2004; Pickering and Gathercole 2001). This finding is surprising, given Evans et al. (2018)’s finding that inhibition and shifting predict spoken word recognition in children with DLD and that updating predicts performance in children with TLD. Interestingly, another study with some of the same children as the current study found that better backwards digit span scores were negatively associated with learning novel talker categories (Levi 2014). In that study, the negative impact of the central executive component was explained in the context of how listeners attempt to categorize talkers’ voices. In particular, children with higher central executive skills may have tried to form explicit rules for categorizing speakers, when processing the speech more holistically (less analytically) would have resulted in better performance. It is less clear how this would apply to the current study. It is possible that children with higher central executive scores were trying to explicitly create a mapping between the accented-speech and stored knowledge about sound production and word production in English (e.g., these speakers eliminate rhotics in coda), whereas a less structured, rigid approach would have been more beneficial (e.g., not all speakers alter rhotics in coda).

Taken together, the current study provides evidence neither for nor against a benefit of exposure to multiple languages during childhood, at least for groups who are matched in several cognitive and linguistic skills. It does, however, provide evidence that linguistic experiences shape how listeners approach tasks and what skills predict better or worse processing of spoken utterances. Future studies will need to further probe the particular components of working memory that support spoken language processing, in light of the simultaneous expected benefit of the forward digit span and the surprising negative impact of the backward digit span.

## Figures and Tables

**Table 1.** Range and means for individual differences measures across the two listener groups and Welch’s *t*-tests for group comparisons.

	Multiple-Variety	Single-Variety	*t*-Value	df	*p*-Value
Age (in months)	80–164 112.3	88–141 112.2	0.01	49.6	0.990
Core Language (CELF)	82–133 101.3	82–123 100.6	0.15	47.2	0.877
PPVT (scaled)	90–144 108.9	78–140 105.8	0.69	50.9	0.491
TONI (scaled)	83–146 109.1	81–150 109.8	−0.13	49.9	0.890
Forward digit (scaled)	5–14 9.36	3–14 8.6	0.99	50.9	0.326
Backward digit (scaled)	5–14 9.7	6–14 9.21	0.71	50.5	0.477
Block span (scaled)	69–143 102.6	55–141 99.0	0.63	50.4	0.529
Recalling Sentences (scaled)	6–17 10.4	6–16 10.36	0.05	48.5	0.958
Phon awareness composite (scaled)	73–118 99.64	76–127 99.68	−0.01	49.5	0.991

**Table 2.** Pearson correlations across all participants. Correlations above 0.50 are bolded.

	Age	PPVT	TONI	Recalling Sentences	Forward Digit Span	Backward Digit Span	Block Span
PPVT	−0.23						
TONI	−0.08	0.28					
Recalling Sent.	−0.01	**0.64**	0.29				
Forward Digit	−0.20	0.39	0.34	**0.60**			
Backward Digit	−0.15	0.21	0.37	0.23	0.33		
Block Span	−0.13	0.07	0.34	0.14	0.13	0.38	
Phon. Awareness	−0.37	**0.56**	0.35	**0.54**	0.48	0.25	0.18

## Data Availability

The data presented in this study are available on request from the corresponding author.

## Appendix A

**Table A1.** Omnibus model output that includes Group. Predictors with *p*-values less than 0.05 are bolded.

Omnibus Model	Estimate	Std. Error	*z*-Value	*p*-Value
**(Intercept)**	−**0.517**	**0.164**	−**3.145**	**0.002**
Group1	−0.089	0.058	−1.547	0.122
**Familiarity1**	**0.801**	**0.109**	**7.363**	**<0.001**
**block1**	−**0.190**	**0.060**	−**3.182**	**0.001**
**scale(age)**	**0.231**	**0.066**	**3.520**	**<0.001**
scale(toni_scaled)	−0.032	0.067	−0.477	0.633
scale(ppvt)	0.018	0.081	0.220	0.826
scale(recall_sent_scaled)	0.178	0.093	1.925	0.054
**scale(forward_digit_scaled)**	**0.165**	**0.078**	**2.122**	**0.034**
**scale(back_digit_scaled)**	−**0.152**	**0.066**	−**2.311**	**0.021**
scale(block_span_scaled)	0.117	0.063	1.856	0.063
scale(PA_scaled)	−0.029	0.077	−0.383	0.702
Group1:Familiarity1	0.023	0.049	0.466	0.641

**Table A2.** Model output for the single-variety exposure group and high familiarity words. Predictors with *p*-values less than 0.05 are bolded.

Single-Variety Exposure/High	Estimate	Std. Error	*z*-Value	*p*-Value
(Intercept)	0.331	0.172	1.925	0.054
block1	−0.168	0.108	−1.549	0.121
**scale(age)**	**0.456**	**0.119**	**3.819**	**<0.001**
scale(toni_scaled)	0.012	0.116	0.103	0.918
scale(ppvt)	0.188	0.129	1.457	0.145
scale(recall_sent_scaled)	−0.076	0.163	−0.467	0.641
**scale(forward_digit_scaled)**	**0.503**	**0.158**	**3.186**	**0.001**
**scale(back_digit_scaled)**	−**0.325**	**0.118**	−**2.763**	**0.006**
scale(block_span_scaled)	0.154	0.103	1.497	0.134
scale(PA_scaled)	−0.034	0.156	−0.219	0.827

**Table A3.** Model output for the single-variety exposure group and low familiarity words. Predictors with *p*-values less than 0.05 are bolded.

Single-Variety Exposure/Low	Estimate	Std. Error	*z*-Value	*p*-Value
**(Intercept)**	−**1.179**	**0.182**	−**6.487**	**<0.001**
block1	−0.247	0.108	−2.292	0.022
scale(age)	0.168	0.119	1.419	0.156
scale(toni_scaled)	−0.148	0.113	−1.316	0.188
scale(ppvt)	0.070	0.127	0.551	0.581
scale(recall_sent_scaled)	0.155	0.158	0.986	0.324
**scale(forward_digit_scaled)**	**0.400**	**0.156**	**2.563**	**0.010**
**scale(back_digit_scaled)**	−**0.281**	**0.119**	−**2.356**	**0.019**
scale(block_span_scaled)	0.060	0.106	0.566	0.571
scale(PA_scaled)	−0.284	0.159	−1.786	0.074

**Table A4.** Model output for the multiple-variety exposure group and high familiarity words. Predictors with *p*-values less than 0.05 are bolded.

Multiple-Variety Exposure/High	Estimate	Std. Error	*z*-Value	*p*-Value
(Intercept)	0.295	0.209	1.411	0.158
**block1**	−**0.241**	**0.122**	−**1.966**	**0.049**
scale(age)	−0.052	0.177	−0.294	0.769
scale(toni_scaled)	0.006	0.139	0.044	0.965
scale(ppvt)	−0.365	0.219	−1.670	0.095
**scale(recall_sent_scaled)**	**0.485**	**0.221**	**2.191**	**0.029**
scale(forward_digit_scaled)	0.067	0.146	0.461	0.645
scale(back_digit_scaled)	−0.177	0.145	−1.221	0.222
scale(block_span_scaled)	0.115	0.142	0.810	0.418
scale(PA_scaled)	0.053	0.135	0.393	0.694

**Table A5.** Model output for the multiple-variety exposure group and low familiarity words. Predictors with *p*-values less than 0.05 are bolded.

Multiple-Variety Exposure/Low	Estimate	Std. Error	*z*-Value	*p*-Value
**(Intercept)**	−**1.356**	**0.200**	−**6.774**	**<0.001**
block1	−0.154	0.122	−1.271	0.204
scale(age)	−0.049	0.174	−0.281	0.779
scale(toni_scaled)	0.046	0.133	0.346	0.729
scale(ppvt)	−0.240	0.207	−1.163	0.245
**scale(recall_sent_scaled)**	**0.438**	**0.217**	**2.022**	**0.043**
scale(forward_digit_scaled)	−0.225	0.145	−1.554	0.120
scale(back_digit_scaled)	−0.126	0.142	−0.887	0.375
scale(block_span_scaled)	−0.027	0.140	−0.193	0.847
scale(PA_scaled)	0.066	0.138	0.477	0.633
